# Marginal Adaptation of Flowable vs Sonically Activated or Preheated Resin Composites in Cervical Lesions

**DOI:** 10.3290/j.jad.b3032461

**Published:** 2022-05-16

**Authors:** Danica Scepanovic, Matej Par, Thomas Attin, Tobias T. Tauböck

**Affiliations:** a Doctoral Student, Department of Conservative and Preventive Dentistry, Center for Dental Medicine, University of Zurich, Zurich, Switzerland. Performed the experiments in partial fulfillment of requirements for a doctoral degree, wrote the manuscript.; b Postdoctoral Researcher, Department of Endodontics and Restorative Dentistry, School of Dental Medicine, University of Zagreb, Zagreb, Croatia. Statistical analysis, visualization, contributed substantially to discussion and writing the paper, proofread the manuscript.; c Chairman, Department of Conservative and Preventive Dentistry, Center for Dental Medicine, University of Zurich, Zurich, Switzerland. Experimental design, proofread the manuscript.; d Head of Division of Cariology and Operative Dentistry, Department of Conservative and Preventive Dentistry, Center for Dental Medicine, University of Zurich, Zurich, Switzerland. Research idea, hypothesis, experimental design, supervision, contributed substantially to discussion and writing the paper, proofread the manuscript.

**Keywords:** preheating, sonic activation, flowable composite, resin composite, marginal adaptation.

## Abstract

**Purpose:**

To investigate marginal integrity of restorations applied with preheated and non-preheated composite, flowable composite, sonically activated composite, and a new thermo-viscous bulk-fill composite using near-infrared technology for preheating, in class V cavities of human molars.

**Materials and Methods:**

Standardized cavities were prepared on the buccal surfaces of 60 human mandibular molars and restored with one of the following resin composite materials after application of an etch-and-rinse adhesive (OptiBond FL, Kerr): non-preheated or preheated conventional composite (Filtek Supreme XTE, 3M Oral Care), preheated thermo-viscous composite (VisCalor bulk, Voco), soncially activated composite (SonicFill 3, Kerr), or flowable composite (Filtek Supreme XTE Flowable, 3M Oral Care) applied in bulk or as a lining material using the snow-plow technique. After light curing and polishing, the percentage of continuous margins (PCM) of the restorations in enamel and dentin was assessed using SEM both before and after thermomechanical loading (TML). TML was carried out with 3000 thermal cycles (5°C–50°C) and a simultaneous mechanical stress application with 1.2 million load-cycles (1.7 Hz, 49 N) in a computer-controlled masticator. Non-parametric statistical analysis was performed using Wilcoxon, Kruskal-Wallis, and Mann-Whitney U-tests (α = 0.05).

**Results:**

All groups revealed a significant decline in marginal integrity after TML in both enamel and dentin. Although the flowable group in enamel and the snow-plow group in dentin showed the highest PCM before TML, the differences between the groups were compensated after TML.

**Conclusion:**

All of the tested composites and application methods showed similar marginal integrities after thermomechanical loading and can be recommended for clinical implementation.

The restoration of class V cavities is a frequently performed clinical dental procedure, but may also be technically challenging, especially regarding the close proximity to the gingiva and difficulties in moisture control.^37^ This might result in poor bonding to the cavity walls and gap formation at the interface between the restoration material and the tooth. Furthermore, shrinkage forces of resin-based composites can induce interfacial microleakage, which may lead to marginal discoloration, secondary caries, or retention loss.^10,27^

Variations in the filler content of resin composites define either sculptable or flowable characteristics of the material. The lower filler load and thus lowered viscosity of flowable composites has been reported to enable better wettability and adaptation to the cavity surface and walls.^5,6,20,35^ Furthermore, the lower elastic modulus (higher elastic capacity) of flowable composites has been associated with a higher degree of flexibility and better resistance to tooth flexure stress at the tooth cervix.^29,55^ However, there are some controversies regarding the effect of flowable composites on marginal adaptation in enamel and dentin. Some studies found no differences in marginal adaptation compared to conventional composites,^2,9^ while others reported better,^49,56^ or even reduced marginal adaptation with flowable composites.^38,41^ A possible explanation might be found in the rather poor mechanical properties of flowable composites due to their reduced filler content.^6^

An approach to improve marginal adaptation of composite restorations is preheating the composite prior to application in the cavity, which reduces its viscosity and temporarily changes the handling characteristic towards that of a flowable composite, while still maintaining enhanced mechanical properties.^23,54^ In addition, increased polymerization temperature enhances monomer conversion, hence resulting in improved physical properties.^4,14,53^ Composite preheating can also reduce shrinkage forces and may therefore improve marginal adaptation.^52^

A further means of reducing the viscosity of resin composites is the use of sonic activation during application, which enables quick placement and improved adaptation to the cavity walls.^3,50^ The moment the sonic activation is stopped, the composite returns to a more viscous consistency, ideal for contouring.^34^ However, it should be noted that sonication is not recommendable for all composite materials and brands, given that increased void formation was observed in some studies.^15,28^

Apart from different composite materials and properties, there are also different insertion techniques.^12^ Opdam et al^36^ suggested the use of the snow-plow technique, which implies the placement of a thin layer of flowable composite as lining material, left uncured and followed by injection of a second layer of a highly viscous composite material. The idea behind this technique is to achieve better wettability of the cavity walls by displacement of the flowable composite when the more viscous composite is applied.^9,36^ However, the effectiveness of reducing microleakage when applying a thin layer of flowable composite has been contentiously discussed.^2,41,57^

With this background, several approaches and techniques to improve the marginal integrity of composite restorations can be taken into consideration. The aim of the present in vitro study was therefore to investigate the effect of a non-preheated composite, a preheated viscous composite, a flowable composite applied in bulk or as lining material using the snow-plow technique, a soncially activated resin composite, and a new thermo-viscous bulk-fill composite using near-infrared technology for preheating, on the marginal integrity of class V restorations before and after thermomechanical loading (TML) in a computer-controlled masticator. The null hypotheses assumed that there would be no differences in marginal integrity: 1. among different restoration approaches before TML, 2. among different restoration approaches after TML, 3. before and after TML for a given restoration approach, and 4. between different dental hard tissues (enamel vs dentin).

## MATERIALS AND METHODS

### Specimen Preparation

Sixty sound human mandibular molars were used in this in-vitro study. The molars were irreversibly anonymized immediately after extraction, stored in refrigerated thymol solution (0.1%), and randomly divided into six groups (n = 10 per group). Only teeth from patients who gave written informed consent prior to the further use of their extracted teeth for research purposes were included. The study complied with the use of anonymized biological material. Therefore, for this study, no authorization from the local ethics committee was required (BASEC request no. 2019-01057).

After cleaning the teeth of remains such as calculus and soft and hard tissue, the apices were sealed (OptiBond FL, Kerr; Orange, CA, USA) to avoid leakage, and one- to two-thirds of the roots were embedded in acrylic resin (Paladur, Heraeus Kulzer; Hanau, Germany). The molars were first affixed to specimen holders with resin composite material (LC Block-Out Resin, Ultradent; South Jordan, UT, USA) and centered with the help of a custom-made device in order to ensure even occlusal loading at a later point in the experiment.

To simulate dentinal fluid pressure,^30^ a power drill (BFW 40/E, Proxxon; Niersbach, Germany) was used to drill a hole slightly below the cementoenamel junction (CEJ) of the distal root reaching the pulp chamber. Pulpal tissue was removed to avoid clogging. A stainless steel tube with a diameter of 1.4 mm was sandblasted, silanized (Monobond Plus, Ivoclar Vivadent; Schaan, Liechtenstein), and then fixated to the drilled hole using an adhesive (OptiBond FL, Kerr) and a flowable composite (Filtek Supreme XTE Flowable Composite, 3M Oral Care; St Paul, MN, USA). Thereafter, the stainless steel tube was connected to a special set-up provided with an infusion bottle and a vacuum pump. In order to generate intrapulpal pressure of 25 mm Hg, the infusion bottle containing physiological saline solution was positioned 34 cm above the specimen. With the aid of a three-way valve, the pulp chamber was first evacuated to achieve a clear, bubble-free state before letting the saline run into it. The intra-pulpal pressure was maintained throughout the whole experiment, starting at least 24 h prior to onset of cavity preparation until completion of thermomechanical loading.

Standardized class V cavities were prepared on the buccal aspects of each tooth with the following dimensions: 3 mm in width, 2.5 mm in height, and 1.5 mm in depth with half of the preparation margin located in enamel and half in dentin. Enamel margins were beveled with a maximum width of 1 mm. For preparation and beveling, an 80-μm cylindrical diamond bur (Universal Prep Set, Intensiv; Grancia, Switzerland) and a 40-μm flame-shaped diamond bur (Universal Prep Set, Intensiv) were used, respectively. The cavities were prepared, checked, and revised if needed using a Galilean loupe (Galilei TTL Sports, ExamVision; Samsø, Denmark) with 2.8X magnification.

### Adhesive Restoration of the Cavities

**Fig 1:**
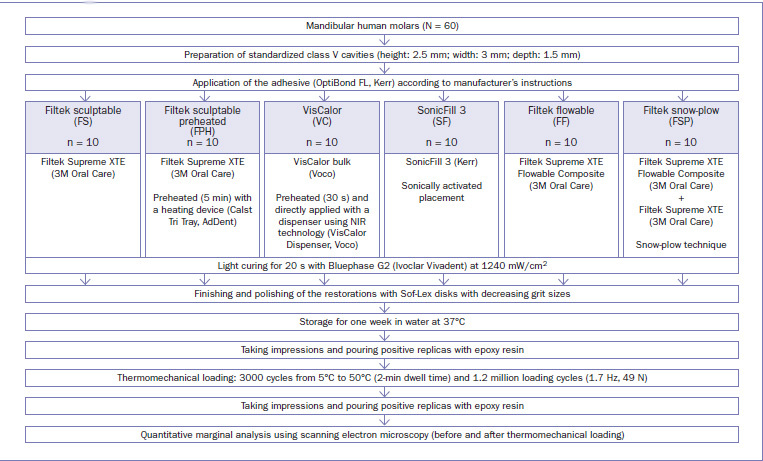
Experimental design.

All cavities were treated with a three-step etch-and-rinse adhesive (OptiBond FL, Kerr) according to the manufacturer’s instructions. After phosphoric acid etching (Ultra-Etch 35%, Ultradent) for 15 s, the cavities were thoroughly rinsed with water and gently air dried. Next, the primer was applied in light scrubbing motions (15 s) and gently air dried (5 s). Thereafter, the adhesive was applied uniformly in a thin layer and light cured (20 s). Light curing was performed using an LED light-curing unit (Bluephase G2, Ivoclar Vivadent; Schaan, Liechtenstein) at a radiant emittance of 1240 mW/cm^2^ immediately adjacent to the restoration surface, ie, at a distance of 0.0–0.5 mm from the cavo-surface margin. The radiant emittance was periodically verified during the experiment using a calibrated radiometer (FieldMax II-TO, Coherent; Santa Clara, CA, USA). After light curing the adhesive, all class V preparations were restored in one increment with different composite materials and filling approaches, as presented in Fig 1. Manufacturers’ information about the materials used are given in Table 1.

**Table 1:** Composition and manufacturers of the materials used

Product	Shade Lot number	Composition	Filler load	Manufacturer	Max. layer thickness
OptiBond FL	N/A 7230984 N/A 7144121	Primer: BHT, CQ, ethanol, GPDM, HEMA, PAMM, water Adhesive: bis-GMA, CQ, GDM, HEMA, ODMAB, barium aluminoborosilicate, Na_2_SiF_6_, fumed silicon dioxide, gamma-methacryloxypropyltrimethoxy-silane	N/A	Kerr; Orange, CA, USA	N/A
VisCalor bulk	A2 1949052	Matrix: amine, BHT, bis-GMA, CQ, dimethacrylate Filler: SiO_2_ nanofillers (20–40 nm), barium-aluminum-silicate glass particles (1.2 μm)	83 wt%	Voco; Cuxhaven, Germany	4 mm
Filtek Supreme XTE	A2B NA44303	Matrix: bis-EMA, bis-GMA, PEGDMA, TEG-DMA, UDMA Filler: non-agglomerated/non-aggregated silica (20 nm) and zirconia (4–11 nm) fillers, aggregated zirconia/silica cluster filler (average cluster particle size: 0.6–10 μm)	78.5 wt% 63.3 vol%	3M Oral Care; St Paul, MN, USA	2 mm
Filtek Supreme XTE Flowable Restorative	A2 NA71771	Matrix: bis-GMA, Procrylat resins, TEG-DMA Filler: YbF_3_ filler (0.1–5.0 μm), non-agglomerated/non-aggregated silica (20 nm, 75 nm), aggregated zirconia (4–11 nm) and silica (20 nm) cluster filler (average cluster particle size: 0.6–10 μm)	65 wt% 46 vol%	3M Oral Care	2 mm
SonicFill 3	A2 695692720	Matrix: bis-EMA, bis-GMA, TEG-DMA Filler: oxides, aluminum, barium glass, silica and YbF_3_ filler (up to 81.5 wt% / 65.9 vol%), inorganic fillers (up to 75 wt% / 55 vol%) with a particle size range of 40 nm–10 μm	81 wt%	Kerr	5 mm

BHT: butylhydroxytoluene; bis-EMA: ethoxylated bisphenol-A-glycidyl methacrylate; bis-GMA: bisphenol-A-glycidyl-dimethacrylate; CQ: camphorquinone; EBADMA: ethoxylated bisphenol-A dimethacrylate; GDM: glycerol dimethacrylate; GPDM: glycerol phosphate dimethacrylate; HEMA: 2-hydroxylethyl methacrylate; ODMAB: 2-(ethylhexyl)-4-(dimethylamino)benzoate; PAMM: phthalic acid monomethacrylate; PEGDMA: poly(ethylen glycol) dimethacrylate; TEG-DMA: triethylene glycol dimethacrylate; TMSPMA, 3-(trimethoxysilyl)propyl methacrylate; UDMA: urethane dimethacrylate.

In the Filtek sculptable (FS) group, a non-preheated composite (Filtek Supreme XTE, 3M Oral Care) was used. In the Filtek sculptable preheated (FPH) group, the same composite as in the FS group was used, but it was preheated for 5 min before application using a heating device (Calset Tri Tray, AdDent; Danbury, CT, USA). In the VisCalor (VC) group, a preheated thermo-viscous composite (VisCalor bulk, Voco; Cuxhaven, Germany) was used. Preheating and application of this composite was performed simultaneously using the VisCalor Dispenser (Voco). The dispenser uses near-infrared technology (NIR) to heat a composite compule in 30 s and provides a constant temperature for a certain period of time (150 s). In both groups where preheated composites were investigated (FPH and VC), fresh compules were used for each cavity. The temperature inside each preheated composite compule reached 65°C and was checked with a digital thermometer (TES-1300, TES Electrical Electronic; Taipei, Taiwan). In the SonicFill 3 (SF) group, a soncially activated composite (SonicFill 3, Kerr; Orange, CA, USA) was used in combination with the SonicFill Handpiece (Kerr). The air-driven handpiece fits a MULTIflex coupler (KaVo Dental; Biberach, Germany) to connect to the dental unit. In the Filtek flowable (FF) group, the cavities were restored with flowable composite (Filtek Supreme XTE Flowable Composite, 3M Oral Care). In the Filtek snow-plow (FSP) group, the snow-plow technique was used. For this purpose, the cavities were initially covered with a thin layer (approximately 0.5 mm) of uncured flowable composite (Filtek Supreme XTE Flowable Composite, 3M Oral Care) followed by a layer of viscous composite (Filtek Supreme XTE, 3M Oral Care) applied on top, displacing the flowable composite to the cavity walls. Before light curing, excess flowable resin composite was carefully removed.

For all six groups, the resin composites were light cured with the same LED light-curing unit used to light cure the adhesive (Bluephase G2, Ivoclar Vivadent; 1240 mW/cm^2^, 20 s). Finishing and polishing were carried out using disks with decreasing grit sizes (Sof-Lex Pop-on, 3M Oral Care) and checked with a microscope (Stemi 1000, Carl Zeiss; Feldbach, Switzerland) at 20X magnification. Afterwards, the specimens were stored in water at 37°C for one week.

### Thermomechanical Loading

Thermomechanical loading was performed using a computer-controlled masticator (CoCoM 2, CPD; Zurich, Switzerland). Thermocycling consisted of 3000 cycles of flushing water with changing temperatures from 5°C to 50°C (2-min dwell time). Mechanical stress was applied simultaneously, with 1.2 million load-cycles transferred to the center of the specimen (1.7 Hz, 49 N). Standardized stainless steel balls (diameter: 1.4 mm) were used as antagonists. The described thermomechanical cycling lasted for 8.2 days^41^ and is considered to simulate five years of clinical service.^8^

### Assessment of Marginal Integrity

Both before and after thermomechanical loading, impressions of the restorations were taken using a silicone elastomer (President Light Body, Coltène; Altstätten, Switzerland). The impressions were then poured with epoxy resin (Epoxyharz L, R&G Faserverbundwerkstoffe; Waldenbuch, Germany), and the obtained positive replicas were glued (Cementit universal, Merz&Benteli; Niederwangen, Switzerland) on aluminum carriers and sputter-coated with gold (SCD 030 Sputter-Coater, Balzers Union; Balzers, Liechtenstein). The margins were quantitatively analyzed using a VEGA TS 5136 XM scanning electron microscope (Tescan Orsay Holding; Brno, Czech Republic) at a standard 200X magnification by a single operator.

Marginal quality was classified as “continuous”, “discontinuous” or “not assessable”, and evaluated both before and after thermomechanical loading. Marginal integrity in enamel or dentin was expressed as the percentage of continuous margins (PCM) in relation to the respective entire assessable margin length.^11^

### Statistical Analysis 

Since Shapiro-Wilk’s test and inspection of normal Q-Q plots indicated that the data significantly departed from normal distribution, non-parametric statistics were used. Pairwise comparisons of PCM measured before and after thermomechanical loading (TML) were performed using the Wilcoxon signed-rank test. Within each combination of substrate (enamel or dentin) and time point (before or after TML), the Kruskal-Wallis test with Dunn’s post-hoc procedure and Bonferroni correction were used to compare PCM between the six experimental groups. PCM between enamel and dentin were compared using the Mann-Whitney U-test. Statistical analysis was performed using SPSS (version 25, IBM; Armonk, NY, USA) at an overall level of significance of α = 0.05.

## RESULTS

**Fig 2:**
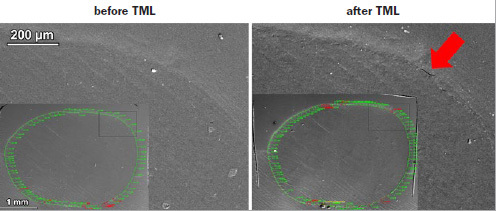
Representative scanning electron micrograph of a class V restoration before and after thermomechanical loading, showing continuous margins (green), discontinuous margins (red), and non-assessable margins (yellow). A magnified detail (black rectangle) shows a perfectly continuous margin before thermomechanical loading, upon which a local discontinuity was observed after thermomechanical loading.

**Fig 3:**
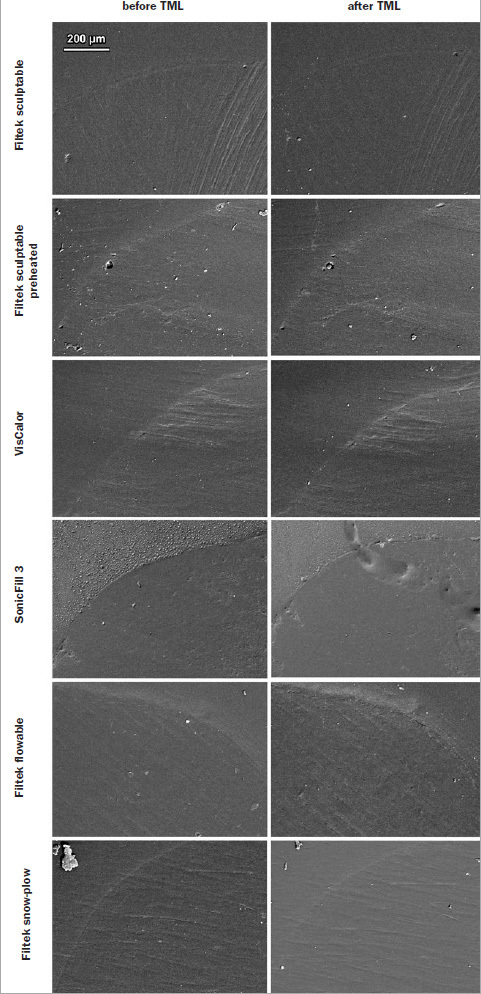
Scanning electron micrographs of restoration margins in enamel before and after thermomechanical loading (TML).

**Fig 4:**
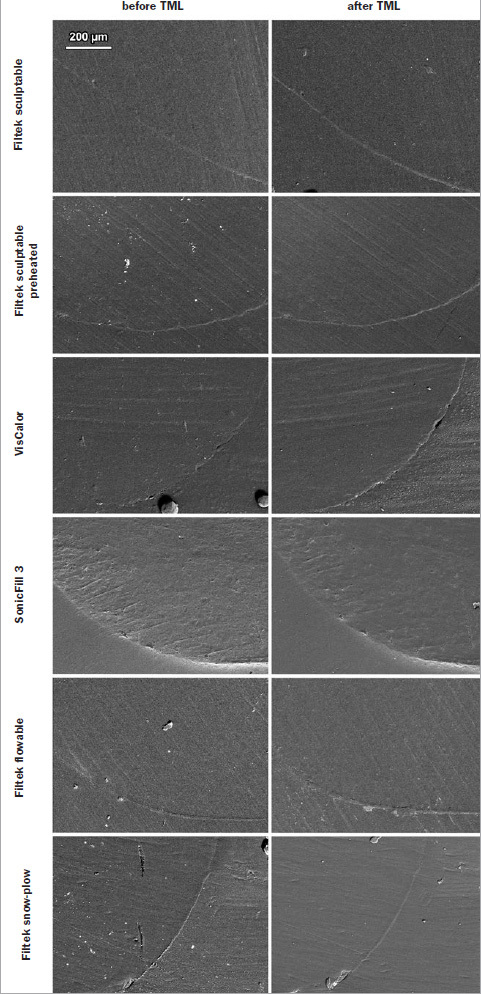
Scanning electron micrographs of restoration margins in dentin before and after thermomechanical loading (TML).

Representative scanning electron micrographs of class V restorations with the designations of margin types (continuous, discontinuous, and not assessable) and localized degradation of restoration margins due to TML are shown in Fig 2. For all experimental groups, the appearance of restoration margins in enamel and dentin is shown in Figs 3 and 4, respectively.

**Fig 5:**
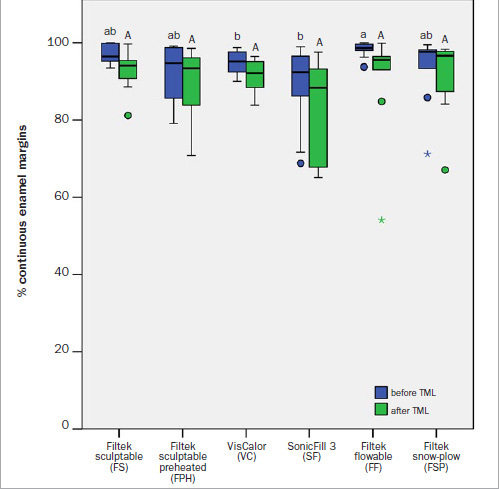
Percentages of continuous enamel margins before and after thermomechanical loading (TML). Same lowercase letters indicate statistically homogeneous groups before TML. Same uppercase letters indicate statistically homogeneous groups after TML. All pairwise comparisons of values obtained before and after TML were statistically significant. The boxplots show the medians (bold black lines), the boxes represent the 25% and 75% data quartiles, whereas the whiskers represent 1.5 x interquartile range (IQR), or minima and maxima of the distribution if below 1.5 x IQR; outliers are represented by circles and extreme outliers by asterisks.

The percentages of continuous enamel margins (%CEM) before and after TML are presented in Fig 5. Pairwise comparisons of %CEM measured before and after TML showed that a significant decrease in %CEM occurred after TML in all six experimental groups (p = 0.005–0.028). Before TML, the FF group showed significantly higher %CEM than did the VC and SF groups (p = 0.022 and 0.032, respectively). In contrast, no significant differences between the groups were found for %CEM after TML.

**Fig 6:**
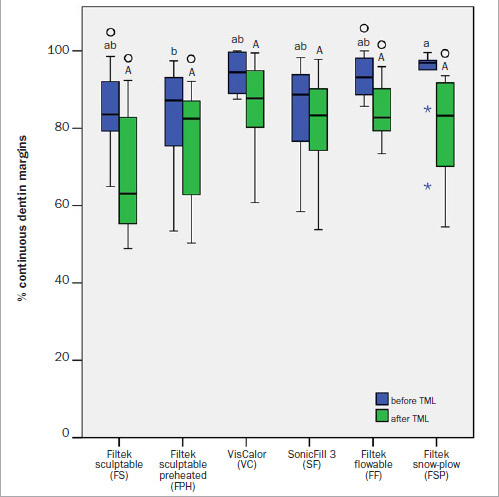
Percentages of continuous dentin margins before and after thermomechanical loading (TML). Same lowercase letters indicate statistically homogeneous groups before TML. Same uppercase letters indicate statistically homogeneous groups after TML. All pairwise comparisons of values obtained before and after TML were statistically significant. Circles (°) above the letters indicate statistically significant differences between enamel and dentin. The boxplots show the medians (bold black lines) and the boxes represent the 25% and 75% data quartiles, whereas the whiskers represent 1.5 x interquartile range (IQR), or minima and maxima of the distribution if below 1.5 x IQR; extreme outliers are presented by asterisks.

The percentages of continuous dentin margins (%CDM) before and after TML are presented in Fig 6. A significant decrease in %CDM was identified after TML for all six experimental groups (p = 0.005–0.007). Before TML, the FSP group had significantly higher %CDM than did the FPH group (p = 0.038). In contrast, the %CDM values obtained after TML showed no significant differences among the groups. The percentages of continuous margins were significantly higher in enamel than in dentin (p = 0.001–0.029), except for FPH and FSP (before TML) and for VC and SF (both before and after TML).

## DISCUSSION

To prevent post-operative sensitivity, marginal discoloration, or secondary caries and to improve marginal integrity and reduce interfacial microleakage of composite restorations, various treatment approaches have been introduced.^42,51^ The present in-vitro study compared different contemporary methods of restorative material placement to evaluate their suitability regarding the marginal integrity of cervical restorations. Significant differences in marginal integrity were identified between the different restoration approaches before thermomechanical loading, which led to the rejection of the first null hypothesis. However, the second null hypothesis could not be rejected, because there were no significant differences between the restoration approaches after thermomechanical loading. As all groups revealed a significant decline in marginal integrity after thermomechanical loading in both substrates, the third null hypothesis was rejected. The fourth null hypothesis, that there would be no difference in marginal integrity between different substrates (enamel vs dentin), was rejected only for some of the experimental groups.

It is well known that the better the adhesion to tooth surfaces, the less likely it is that gaps will form at restoration margins due to polymerization shrinkage and mastication forces.^1,13^ However, no correlation has been established between bond strength and marginal adaptation in dentin, thus indicating that mechanical properties of the adhesive interface are crucial for resilient adhesion.^8^ In this study, an established multi-step etch-and-rinse adhesive system (OptiBond FL, Kerr) was used, which showed good results with predictable adhesion in both laboratory and clinical studies.^22,32,44^ As shown by recent meta-analyses,^17,18^ the outcomes of the bonding procedure depend on multiple factors, one of which is the choice of bonding approach, that cannot be easily identified as a decisive factor for the retention and marginal quality of a restoration. These conclusions are especially evident when comparing the results of different research groups that differ in the choice of adhesive, operative protocol, aging protocol, and evaluation criteria. In the present study, this issue was addressed by consistently applying a single bonding system by a single trained operator and performing restorations in a random order. Thus, irrespective of the investigated composite application techniques, the adhesive used in the present study contributed to marginal integrity (enamel/dentin) above 95%/90% before TML and 93%/80% after TML.

Quantitative marginal analyses are performed under various experimental conditions, as no standard protocol has been established for cavity design^25^ or artificial aging simulation.^31^ Most commonly, class II or class V restorations in extracted human teeth have been used and subjected to various artificial aging protocols through thermal or thermomechanical cycling.^21,24^ For class V cavities, two well-known protocols named after their respective research institutions (“Berlin” and “Zurich”) were described and critically evaluated by Heintze et al.^26^ The described protocols differed in terms of cavity size and artificial aging simulation. The “Berlin” protocol used slightly larger cavities (apical-coronal dimension: 4 mm; mesial-distal dimension: 3 mm; depth: 1.5 mm), and its aging simulation consisted of 2000 thermocycles between 5°C and 55°C, without mechanical loading. In comparison, the “Zurich” protocol used 3000 thermocycles and an additional 1.2 million mechanical load-cycles of 49 N. Although the “Zurich” protocol used in this study is nominally well-standardized, the quantitative results obtained in the present study were analyzed only internally, without attempting to compare them to external data reported in other studies. The main reason is that the quantitative margin analysis is known to be strongly operator-dependent regarding both the preparation of cavities and the evaluation of margins on SEM micrographs; the operator/evaluator bias can be responsible for up to 20% of the variation.^26^ To minimize this type of bias, all cavities in the present study were prepared in random order by a single trained dentist. The same dentist was also trained and calibrated to perform the SEM evaluation of margins.

In the present study, a significant decrease of marginal integrity in enamel and dentin was found after thermomechanical loading in all experimental groups. Furthermore, significantly lower marginal integrity was revealed in dentin compared to enamel, with the exception of the following groups: FPH and FSP (before thermomechanical loading), and VC and SF (both before and after thermomechanical loading). For VC and SF, marginal integrities were similar in enamel and dentin before and after thermomechanical loading, indicating the potential of preheated and soncially activated composites for improved resistance to the challenges of dentinal adhesion even after thermomechanical loading. A challenge and a likely cause of the inferior marginal integrity of the restorations in dentin identified in most of the experimental groups might be the application of pulpal pressure and thus perfusion of fluid through dentin tubules, which is detrimental to the adhesive-dentin interface.^30,48^ The simulation of pulpal pressure and artificial aging by thermomechanical loading are commonly used to simulate the composite/tooth adhesive interface in bond strength tests^45^ and marginal integrity evaluations.^26^ Although there is some evidence that the simulation of pulpal pressure does not improve the correlation of in-vitro bond strength data with clinical results,^45^ it was used in the present study as a standard part of the experimental protocol.^30^ Another challenge to attaining adhesion to dentin is its composition: it has a higher organic content than does enamel. Dentin mainly consists of collagen fibrils capable of forming a hybrid layer with the adhesive.^22^ This micromechanical interlocking may be hindered by bur preparation, which results in the formation of a smear layer.^51^ To remove this smear layer, an etch-and-rinse adhesive was used in this study. However, removal of the smear layer with phosphoric acid etching may in turn increase dentinal fluid flow and could thus additionally deteriorate marginal adaptation.^43^ Furthermore, after acid etching, endogenous matrix metalloproteinases bound to the dentin organic matrix can degrade exposed dentinal collagen fibrils within the hybrid layer, if insufficiently impregnated with adhesive monomers.^33^

Another influencing factor for marginal integrity is the ability of the restorative materials to wet the cavity surfaces.^9^ In the present study, the FF group revealed significantly better marginal adaptation in enamel compared to VC and SF before thermomechanical loading. In dentin, the FSP group showed significantly better marginal adaptation compared to FPH, also before thermomechanical loading. The better performance of restoration modalities that use flowable composites can be attributed to the lower filler load of flowable composites compared to sculptable composites, which enables their flowable character and thus greater wettability.^47^ At the same time, it has been shown that a lower filler load may be disadvantageous in terms of mechanical properties of materials and shrinkage forces.^16,39,40^ This might have contributed to the results obtained after thermomechanical loading, with apparently no significant differences in marginal integrity among the various restoration modalities.

Considering the data scattering within individual experimental groups, it can be seen that interquartile ranges (IQRs) for both dental substrates increase after thermomechanical loading. This finding suggests that the marginal quality of restorations made from the tested composite materials becomes more unpredictable as the restorations age. For the FSP group, the significant decrease of marginal integrity after thermomechanical loading and the accompanying increase in data scattering might be explained by the heterogeneous displacement of the underlying flowable composite for the snow-plow technique. Furthermore, shrinkage forces of the overlying composite during photopolymerization might pull the uncured flowable composite from the cavity walls and cause localized marginal discontinuity.

The significantly lower initial marginal integrity of SF compared to FF in enamel might be explained by the comparatively higher elastic modulus of SF, which might have caused microfracturing at restoration margins due to higher stress formation.^7,19,50^ However, after thermomechanical loading, SF showed marginal adaptation that was statistically similar to the other composite materials. Clinical data also showed no differences in success rates between soncially activated and conventional restorative composite.^3^

Along with sonic activation, increasing the temperature of composites during application enables enhanced adaptation to the cavity walls by reducing the material’s viscosity.^15^ Additionally, preheated composites have shown significantly lower shrinkage forces at an improved degree of conversion.^14,52^ However, it should be taken into consideration that the temperature of the composite drops rapidly upon removal from the heating device.^14,46^ To prevent cooling of the composite, a novel all-in-one device (VisCalor Dispenser, Voco) employing near-infrared technology was used in the VC group. The composite compules can be warmed up and applied immediately after preheating using the VisCalor Dispenser without the need of first removing the device from the warmer. Additionally, the dispenser makes it possible to maintain a constant composite temperature during the entire application process, which helps to prevent a drop in temperature during application. This approach, however, could not enhance marginal integrity of the class V composite restorations in the present study.

Last but not least, as commercial composites differ in their mechanical properties and polymerization shrinkage behavior, all studies on properties affected by shrinkage-related variables are necessarily disadvantaged by the fact that the material’s compositional details are mostly unknown. Unlike investigations of experimental materials, which allow individual parameters to be adjusted according to the researcher’s desires, in studies on commercial composites, the individual outcomes are difficult to attribute to particular material characteristics, as the latter are only incompletely known to the investigators. The present study tried to simulate a clinical placement of contemporary commercial composites in class V cavities using a consistent restorative procedure performed by a single trained operator, in an attempt to identify whether different restorative approaches yield differences in marginal integrity. In this regard, no generalizations to other combinations of composite materials and adhesives should be made, as only the resultant behavior of individual materials and restorative approaches was observed, with no insight into contributions of individual fundamental material variables.

## CONCLUSION

Although restoration approaches using flowable composite (alone or as part of the snow-plow technique) initially showed slightly better marginal integrity compared to restoration techniques using preheated or soncially activated composites, thermomechanical loading led to similar marginal integrity for all investigated restoration approaches. All of the investigated restoration approaches using sculptable, flowable, preheated, or soncially activated composites performed similarly with regard to marginal integrity of class V composite restorations and can be considered appropriate for clinical implementation.

## Clinical relevance:

Sculptable, flowable, preheated, and sonically activated composites perform similarly with regard to the marginal integrity of class Vcomposite restorations and are suitable for clinical implementation.
